# TNK Window into His Brain: If This Aphasic Veteran Could Talk

**DOI:** 10.1007/s11606-025-09553-z

**Published:** 2025-04-29

**Authors:** Lealani Mae Y. Acosta

**Affiliations:** Department of Neurology, Vanderbilt University Medical Center, Nashville, TN USA

This is a fictional piece that imagines a “conversation” between a patient experiencing a stroke that renders him aphasic, though we can read his thoughts, and the team of doctors assessing his symptoms as part of a stroke alert. The protocol of questions and examination determines whether he is a candidate for tenectaplase (TNK), intervention for acute ischemic stroke.

“What’s your name? How old are you?”

My Lulu called me Jackie when she was alive. Junior calls me Pops. The boys at the barbershop call me Rooster because they’re jealous of my hair. You can call me Mr. Jackson, son.

“Did you make out any of what he said? Sounds like gibberish to me.” She whips out a tablet deep from her white coat pocket to document the exam.

I can hear you just fine. Can’t you hear me, doc? Why won’t my lips move?

“Close your eyes!” He peers into Mr. Jackson’s eyes with a penlight.

Yeah, I blinked. You so darn close, you spit in my eye!

“Raise your right arm! One, two, three …” He continues to bark commands.

“He’s definitely got some effort.” She nods approvingly.

Could’ve crushed your fingers when I was younger, son. Spent more years than you been alive ratcheting socket wrenches. Why can I move my left, but not my right? Stupid arm. Arms near froze in place clutching my rifle in Vietnam. Wading down rivers, walking through jungle, praying if I got tangled up, it was only vines and not a damn (sorry, Lulu) snake. Nightmares still wake me up, sheets damp like I just stepped out that river. “You’re okay, honey. Go back to sleep,” my Lulu would say. She’d hum a hymn, stroke my forehead. Her hands were so soft.

“How many fingers am I holding up?”

I’ll give you a finger, son. I’m looking at you. With my stink eye.

“Anything in his chart about a previous Bell’s? His frontalis isn’t activating. Can you find his driver’s license?”

What in the Sam Hill you doing with my wallet?

“His left arm is definitely functional!” She holds his billfold beyond his extended grasp.

“One, two, three, four, five, six, seven … Keep that wallet out of reach. Does he have a droop in the photo?” he asks while pulling out a reflex hammer.

Young lady, get me my wallet. Ain’t got no license. Stopped after my wreck.

Is that INR back? Chart says afib and there’s an old script for warfarin, but it hasn’t been renewed in two years.

When my Lulu was here, she kept my appointments. Now she’s gone, ain’t no good trying. Junior moved in. He’s a good boy, but he’s no Lulu. Where is Junior?

My Lulu. Always fussing in her pink robe. Glasses sliding down her little nose. She filled my pill box. Bitty red ones and big fat horse pills in one hand, mug of black coffee in the other. Never needed sugar with my Lulu. “Drink up, honey, we gotta stay healthy together!”.

“Can you feel this?”.

Sure enough! Wish I could talk. I’d give you a piece of my mind. Wait … why can’t I feel you pinching me?

Is that repeat glucose back? What was it in the field: 420? Did he get insulin?

Last I remember was that powdered donut. My Lulu never let me. Junior knows it makes me happy. Love licking the sugar off my fingers, otherwise the remote’s too sticky. Westerns on TV, ice cold beers in the fridge, donuts by my easy chair. My Lulu always did the cooking. “Pops, you know I can’t cook worth a darn.” Bless his heart. Junior tries.

“Show me your teeth! Smile!”.

You want my teeth? Y’all can find them in a glass in my bathroom.

“Lift your left leg! Touch my hand with your foot! One, two, three, four, five. Take it easy, sir!”

I’d like to kick you in the britches, son. Why you still hollering?

“Lift your right leg! One, two …”

“At least he’s trying.”

Can’t rightly get that right leg to work. Like that night. Stupid leg. Driving home from Bubba and Hattie’s. Didn’t think it’d been raining that hard, but never saw the bridge like that. Lulu, you always used to say, “The good Lord willing and the crick don’t rise.” Well, the crick rose. Kept trying to pump the brake, but my foot wouldn’t work. Pulled that wheel with all my might, but it wasn’t enough. Last thing I remember was you screaming.

Woke up in this hospital. Hate this blue wallpaper. Hate these thin blue curtains; ain’t nobody got no privacy. Hate those smiley face signs telling me to wash my hands. Doctors said I had a … a … P-I-A. All I know it’s all been a pain in the … well, you know, Lulu. Almost had a stroke. Everybody kept saying how blessed I was to be alive. Wish I’d died. Least I held your hand at the end.

Lulu, I’m sorry, baby. Said I’d always take care of you. Wanted to rip out those tubes and put them in me instead. Wanted to rip out those tubes so I could hold you. I begged those doctors. Begged the good Lord to take me instead. I cussed everybody out. I’m sorry, baby: I know you didn’t like that. You’d wrinkle your little nose and give me that look you always gave Junior when he had his hand in the cookie jar. Mercy, I’d give anything for you to be back and give me that stink eye again.

“Do you see that bright signal? Remember that. That’s pretty classic for a hyperdense left middle cerebral artery sign.”

You saw the good in people, Lulu. Always had faith, trust. I know I ain’t been the same since Nam. “Stop smoking, stop drinking.” Ain’t nothing else to dull the pain. I did better when my Lulu was alive. When the doctors couldn’t save her, I gave up on them too.

Can you try reaching his son again to confirm last known normal? When was he found down?

Son, I may be down, but don’t count me out. This ain’t my first rodeo. If my Lulu had seen me like this … I swear, Lulu, if I get through this, I promise I will do better. For you.

“What about the TNK?”.

